# Effects of Nitric Oxide on Voltage-Gated K^+^ Currents in Human Cardiac Fibroblasts through the Protein Kinase G and Protein Kinase A Pathways but Not through *S*-Nitrosylation

**DOI:** 10.3390/ijms19030814

**Published:** 2018-03-12

**Authors:** Hyemi Bae, Jeongyoon Choi, Young-Won Kim, Donghee Lee, Jung-Ha Kim, Jae-Hong Ko, Hyoweon Bang, Taeho Kim, Inja Lim

**Affiliations:** 1 Department of Physiology, College of Medicine, Chung-Ang University, 84 Heukseok-ro, Seoul 06974, Korea; hyemiworld@cau.ac.kr (H.B.); cju415@cau.ac.kr (J.C.); stream00@cau.ac.kr (Y.-W.K.); ldh8606@cau.ac.kr (D.L.); akdongyi01@cau.ac.kr (J.-H.K.); haena@cau.ac.kr (H.B.); 2 Department of Family Medicine, College of Medicine, Chung-Ang University Hospital, 102 Heukseok-ro, Seoul 06973, Korea; girlpower219@cau.ac.kr; 3 Department of Internal Medicine, College of Medicine, Chung-Ang University Hospital, 102 Heukseok-ro, Seoul 06973, Korea

**Keywords:** delayed rectifier K^+^ channel, human cardiac fibroblasts, nitric oxide, protein kinase A, protein kinase G, *S*-nitrosylation, transient outward K^+^ channel, voltage-gated K^+^ channels

## Abstract

This study investigated the expression of voltage-gated K^+^ (K_V_) channels in human cardiac fibroblasts (HCFs), and the effect of nitric oxide (NO) on the K_V_ currents, and the underlying phosphorylation mechanisms. In reverse transcription polymerase chain reaction, two types of K_V_ channels were detected in HCFs: delayed rectifier K^+^ channel and transient outward K^+^ channel. In whole-cell patch-clamp technique, delayed rectifier K^+^ current (I_K_) exhibited fast activation and slow inactivation, while transient outward K^+^ current (I_to_) showed fast activation and inactivation kinetics. Both currents were blocked by 4-aminopyridine. An NO donor, *S*-nitroso-*N*-acetylpenicillamine (SNAP), increased the amplitude of I_K_ in a concentration-dependent manner with an EC_50_ value of 26.4 µM, but did not affect I_to_. The stimulating effect of SNAP on I_K_ was blocked by pretreatment with 1H-(1,2,4)oxadiazolo[4,3-a]quinoxalin-1-one (ODQ) or by KT5823. 8-bromo-cyclic GMP stimulated the I_K_. The stimulating effect of SNAP on I_K_ was also blocked by pretreatment with KT5720 or by SQ22536. Forskolin and 8-bromo-cyclic AMP each stimulated I_K_. On the other hand, the stimulating effect of SNAP on I_K_ was not blocked by pretreatment of *N*-ethylmaleimide or by DL-dithiothreitol. Our data suggest that NO enhances I_K_, but not I_to_, among K_V_ currents of HCFs, and the stimulating effect of NO on I_K_ is through the PKG and PKA pathways, not through *S*-nitrosylation.

## 1. Introduction

Human cardiac fibroblasts (HCFs) are the highest cell population in the myocardium, accounting for approximately two-thirds of the cells [1], and play a role in cardiac development, myocardial structuring, cell signaling, and electro-mechanical function in healthy and diseased myocardium [2]. Although HCFs are not electrically excitable, they express a plethora of ion channels that modulate cardiac electrical function. The distribution and properties of their ion channels are distinct from those of cardiomyocytes [3]. The electrical coupling between fibroblasts/myofibroblasts and ventricular cardiomyocytes has been demonstrated not only in co-culture conditions [1,4,5], but also in the whole heart [6]. In fact, the two types of cells can directly couple to each other via connexin-based gap junction [7]. High rate of electrical activation from tachy-paced atrial cardiomyocytes induce them to secret factors into the culture medium that in turn activate fibroblasts [8]. Conversely, cardiac fibroblasts paracrine factors alter impulse conduction and ion channel expression of cardiomyocytes [9]. In addition, these interactions are enhanced in response to cardiac injury [10]. 

Voltage-gated K^+^ (K_V_) currents are activated on membrane depolarization, regulation of resting membrane potential, influence the amplitudes and durations of myocardial action potentials, and the electrical coupling between the cells and cardiac fibroblasts [11,12]. Mainly, two classes of K_V_ currents have been distinguished based primarily on differences in time- and voltage-dependent properties: slowly inactivating delayed rectifier K^+^ current, referred to as I_K_ and rapidly inactivating transient outward K^+^ current, I_to_, which can be differentiated by electrophysiological and pharmacological studies [3,13,14,15]. The two types of K_V_ currents are also present in HCFs [3] but the molecular determinants underlying I_K_ and I_to_ have not been extensively studied. 

Nitric oxide (NO), a ubiquitous cellular messenger, is synthesized by essentially all cardiac cell types and plays a key role in the regulation of cardiac functions [16,17], including the direct modulation of myocardial contractility [18], myocardial regeneration [19], hypertrophic remodeling [20], and apoptosis [21]. These effects of NO are related to the modulation of the activity of cardiac ion channels implicated in the generation of cardiac action potential [22]. Previous reports demonstrated that NO inhibits I_K_ in mouse ventricular myocytes [23], but increases this current in guinea-pig ventricular myocytes [24]. In addition, NO inhibits I_to_ in human atrial and ventricular myocytes [25]. 

NO exerts its biological effects through cyclic GMP (cGMP)-dependent and cGMP-independent signaling pathways. Specificity of cGMP signals is achieved through cGMP synthesis by soluble guanylate cyclase (sGC). In cardiomyocytes, the physiological effects of cGMP are exerted through the activation of protein kinase G (PKG). Increasing evidence suggests that cGMP-dependent signaling pathways play an important role in inhibiting cardiac remodeling and represent a promising therapeutic target for treatment of cardiovascular diseases [26]. 

Although sGC was the first identified receptor for NO, NO exerts a ubiquitous influence in a cGMP-independent manner. NO can modulate the cAMP/protein kinase A (PKA) signaling pathway; low levels of NO increase cAMP, by activation of adenylate cyclase (AC) in rat ventricular myocytes [27]. In addition, many effects of NO are mediated by *S*-nitrosylation, the covalent modification of a protein cysteine thiol by an NO group to generate an *S*-nitrosothiol that implicates in all major functions of NO in the cardiovascular system [28,29,30]. 

However, the effects of NO on K_V_ channels of HCFs and the underlying cyclic nucleotide mechanisms remain unclear. Therefore, this study aimed to analyze the effects of NO on K_V_ channels in cultured human ventricular fibroblasts, as well as the intracellular signaling pathway responsible for these effects.

## 2. Results

### 2.1. Identification of Two Types of Voltage-Gated K^+^ Channels in Human Cardiac Fibroblasts by RT-PCR

**Figure 1:**
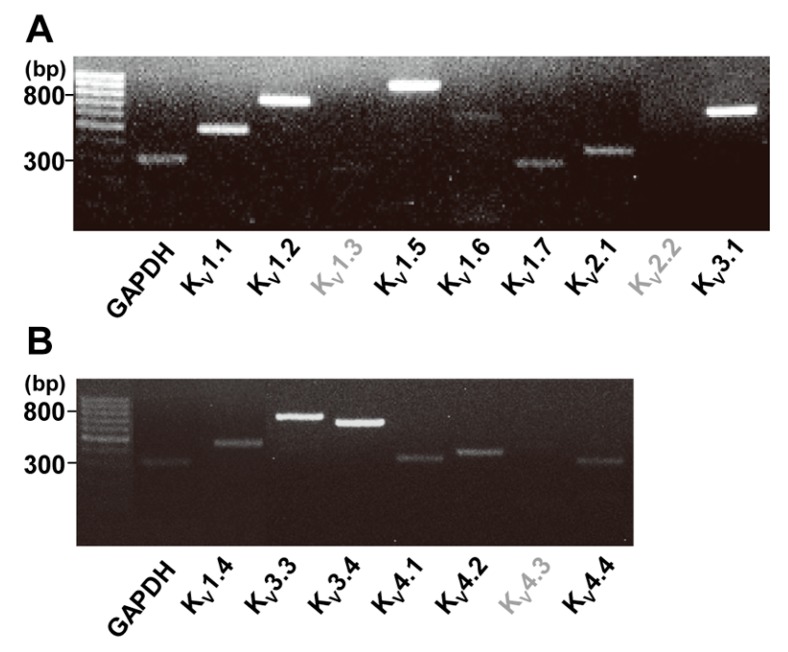
Identification of two types of voltage-gated K^+^ (K_V_) channels in human cardiac fibroblasts by reverse transcription polymerase chain reaction (RT-PCR). (**A**) Strong gene expression was shown for K_V_1.1, K_V_1.2 K_V_1.5, and K_V_3.1 for delayed rectifier K^+^ (I_K_) channels and (**B**) K_V_3.3 and K_V_3.4 for transient outward K^+^ (I_to_) channels_._ Weak expression was observed for K_V_1.6, K_V_1.7 and K_V_2.1 (for I_K_) and K_V_1.4, K_V_4.1, K_V_4.2, and K_V_4.4 (for I_to_). Glyceraldehyde 3-phosphate dehydrogenase (GAPDH) was used as a positive control.

In the present study, we used RT-PCR targeting human genes for K_V_ channel pore-forming α subunit in HCFs. The expressions of important genes for K_V_ currents in cardiac myocytes, the pore-forming α subunits of K_V_1 (Shaker) family, K_V_2 (Shab) family, K_V_3 (Shaw) faimly, and K_V_4 (Shal) faimly were tested [14]. Strong gene expression was observed with K_V_1.1, K_V_1.2, K_V_1.5, and K_V_3.1, while weak gene expression was exhibited with K_V_1.6, K_V_1.7, and K_V_2.1 (Figure 1A). All of the products of these genes are parts of I_K_ channel. For the I_to_ channel, strong gene expression of K_V_3.3 and K_V_3.4 and weak expression of K_V_1.4, K_V_4.1, K_V_4.2, and K_V_4.4 were seen in the HCFs (Figure 1B). Gene expression of K_V_1.3, K_V_ 2.2 (for I_K_ channel) and K_V_4.3 (for I_to_ channel) were not observed in the HCFs. 

### 2.2. Identification of Two Types of Voltage-Gated K^+^ Channels using Electrophysiological Methods

**Figure 2:**
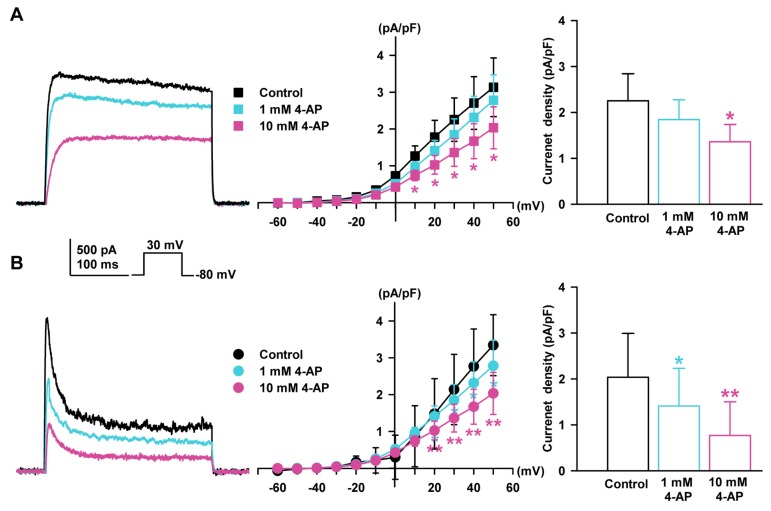
Effect of 4-aminopyridine (4-AP) on K_V_ currents in HCFs. (**A**) The representative rapid activated and slow inactivated, I_K_ recorded in a single HCF in control conditions and after application of 4-AP (1 or 10 mM). The current-voltage (*I-V*) relationship of steady-state currents with I_K_ changed in the presence of 4-AP, and the concentration-response bar graphs for the 4-AP effect on I_K_ are shown. (**B**) Typical rapid-activated and inactivated I_to_ in control and after 4-AP (1 or 10 mM) addition. The *I-V* relationship of peak currents changed by the presence of 4-AP on I_to_ and concentration-response bar graphs are shown. * *p* < 0.05, ** *p* < 0.01 versus the control.

The two types of K_V_ currents with typical behavior were recorded (I_K_ and I_to_) with the whole-cell patch clamp technique. The stimulation voltage protocol consisted of depolarizing steps repeated every 10 s with a 10-mV increment for 400 milliseconds (−80 mV of holding potential). To exclude the influence of large-conductance Ca^2+^-activated K^+^ current (IBK_Ca_), which is another major K^+^ current, iberiotoxin (100 nM) was added to the bath solution and EGTA (10 mM) into the pipette solutions. These currents could be distinguished based on their activation and inactivation kinetics. I_K_ exhibited fast activation and slow or partial inactivation (Figure 2A) and I_to_ demonstrated fast activation and inactivation kinetics (Figure 2B).

I_K_ and I_to_ were detected in 82.5% (*n* = 312 of 378) and 17.5% (*n* = 66 of 378) of the cells, respectively. These currents could also be distinguished based on the effects of K^+^ current blockers. Both K_V_ currents were sensitive to 4-aminopyridine (4-AP). I_K_ was sensitive to a high concentration of 4-AP (10 mM, −39.6 ± 6.4% of the control, in steady state current at +30 mV, *n* = 4, *p* < 0.05, Figure 2A) and I_to_ was sensitive to a lower concentration of 4-AP (1 mM, −30.7 ± 8.6% of the control, *n* = 4, *p* < 0.05; 10 mM, −62.3 ± 7.7% of the control, in peak current at +30 mV, *n* = 4, *p* < 0.01, Figure 2B). 

**Figure 3:**
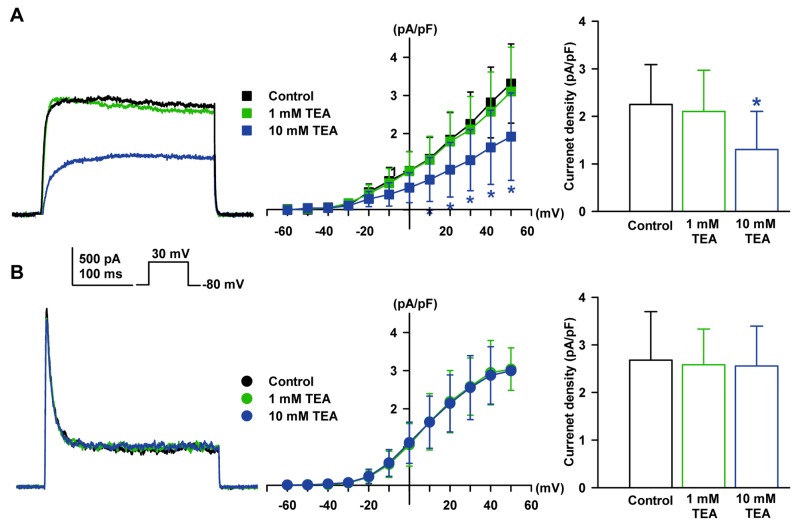
Effect of tetraethylammonium chloride (TEA) on K_V_ currents in HCFs. (**A**) The representative I_K_ recorded in a single HCF in control conditions and after application of TEA (1 or 10 mM) are shown. The *I-V* relationship of the steady-state current change of I_K_ by TEA and bar graphs for concentration change are also shown. (**B**) The typical I_to_ was not changed by TEA. The *I-V* relationship of the peak current change for the TEA of I_to_ and bar graph for concentration response are shown. * *p* < 0.05 versus the control.

On the other hand, when assessing the effects of tetraethylammonium chloride (TEA), another K^+^ channel blocker, I_K_ was inhibited at a high concentration of TEA (10 mM, −42.1 ± 9.6% of the control, *n* = 4, *p* < 0.05, Figure 3A) but not at a low concentration of TEA (1 mM). However, I_to_ was not inhibited by 10 mM TEA (−4.5 ± 8.2% of the control, *n* = 4, Figure 3B).

### 2.3. Effect of NO on Two Types of Voltage-Gated K^+^ Currents

**Figure 4:**
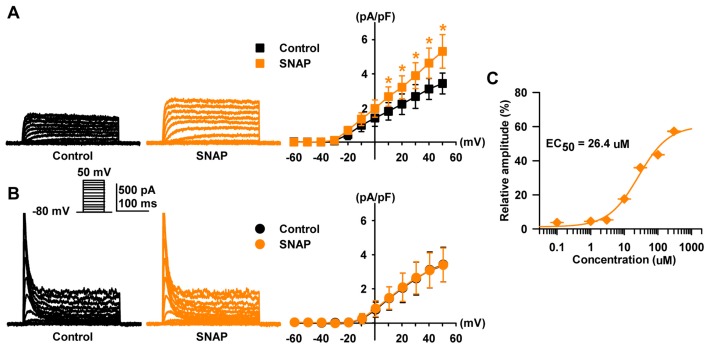
Effect of nitric oxide (NO) on two subtypes of K_V_ currents. Raw data and the *I-V* relationship of steady-state currents changed by the presence of SNAP on (**A**) delayed rectifier K^+^ current (I_K_) and (**B**) transient outward K^+^ current (I_to_). (**C**) The concentration-response curve of S-nitroso-N-acetylpenicillamine (SNAP) on I_K_. The continuous line represents the fit to the Hill equation. The normalized currents (percent inhibition) were calculated from the I_K_ in the absence of SNAP and plotted against various concentrations of SNAP. * *p* < 0.05 versus the control.

To determine the effect of NO on K_V_ currents in HCFs, SNAP (an NO donor, 100 µM) was added to the bath solution. The amplitude of I_K_ was significantly increased by SNAP (+46.0 ± 10.9% of the control, *n* = 6, *p* < 0.05, Figure 4A). On the other hand, I_to_ was not activated by SNAP (+1.6 ± 9.8% of the control, *n* = 6, Figure 4B). After the addition of SNAP, the current density of I_K_ at +30 mV increased from 2.67 ± 0.70 pA/pF to 3.89 ± 0.76 pA/pF. On the other hand, the current density of I_to_ in the peak state was not altered by SNAP (2.56 ± 0.96 pA/pF to 2.60 ± 0.94 pA/pF). 

Various concentrations of SNAP (0.1 to 300 µM) were applied and the I_K_ was then examined to explore whether SNAP-induced stimulation was concentration dependent (Figure 4C). I_K_ was elicited by one-step depolarizing pulses of +30 mV. The I_K_ was increased by increasing concentrations of SNAP, but the low level of NO observed in the physiologic condition did not affect the I_K_ of HCFs. Steady-state currents normalized by control data were fitted with the Hill equation, producing an EC_50_ value of 26.4 µM and a Hill coefficient of 0.96 (*n* = 7).

### 2.4. Effect of NO on Delayed Rectifier K^+^ Current through PKG Signaling Pathway

**Figure 5:**
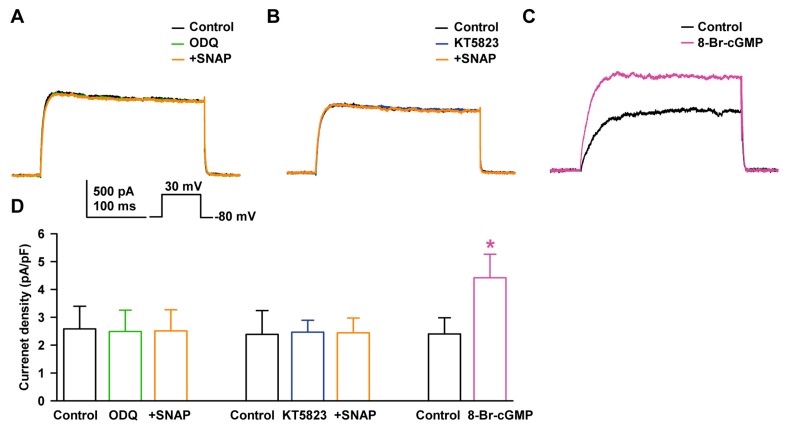
Effects of the PKG pathway on the stimulating effect of NO on I_K_. Representative currents showing the effect of SNAP on I_K_ after pretreatment with (**A**) 1H-(1,2,4)oxadiazolo[4,3-a]quinoxalin-1-one (ODQ) or (**B**) KT5823. (**C**) Effect of 8-Br-cyclic GMP on I_K_. (**D**) Bar graph summarizing current density changes for the effects of SNAP on I_K_ after pre-incubation with ODQ or KT5823, and the effect of 8-Br-cyclic GMP on I_K_ current. * *p* < 0.05 versus the control.

NO activates sGC and produces cGMP, which activates PKG. We examined the contribution of cGMP to SNAP-induced I_K_ enhancement, using 1H-(1,2,4)oxadiazolo[4,3-a]quinoxalin-1-one (ODQ, a sGC blocker). When the cells were pretreated with ODQ (10 µM), I_K_ did not increase in the presence of 100 µM SNAP (–2.6 ± 9.3% of the control, *n* = 7, Figure 5A). To further confirm the contribution of the cGMP signaling pathway, KT5823 (a PKG blocker, 1 µM) was added to the bath, and SNAP then failed to increase I_K_ (+2.3 ± 6.2% of the control, *n* = 7, Figure 5B).

We also assessed the effect of cGMP, which is generated from NO binding to sGC. 8-Bromo-cyclic GMP (8-Br-cGMP, an activator of PKG, 300 µM) increased I_K_ to +84.0 ± 14.5% of the control (*n* = 4, *p* < 0.05, Figure 5C). The current densities at +30 mV with the addition of SNAP were not altered after ODQ pretreatment (2.58 ± 0.81 pA/pF at control, 2.49 ± 0.77 pA/pF at ODQ, 2.51 ± 0.75 pA/pF at SNAP, Figure 5D) or after KT5823 pretreatment (2.39 ± 0.85 pA/pF at control, 2.47 ± 0.42 pA/pF at KT5823, 2.44 ± 0.53 pA/pF at SNAP). On the other hand, the current density with 8-Br-cGMP treatment increased significantly (from 2.40 ± 0.58 pA/pF to 4.42 ± 0.84 pA/pF).

### 2.5. Effect of NO on Delayed Rectifier K^+^ Current through PKA Signaling Pathway

**Figure 6:**
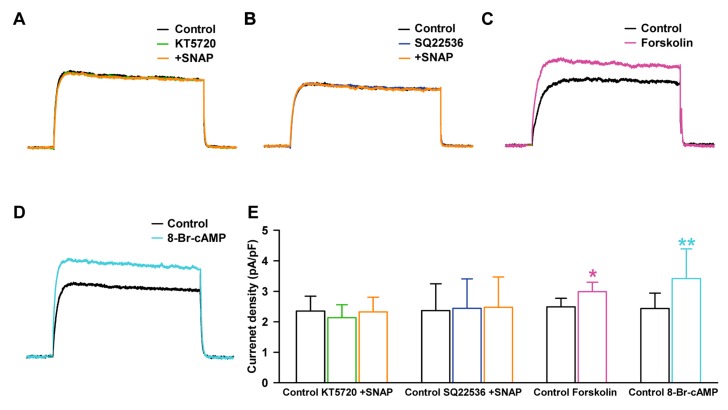
Effects of the protein kinase A (PKA) pathway on the stimulating effect of NO on I_K_ current. Representative currents showing the effect of SNAP on I_K_ after pretreatment with (**A**) KT5720 or (**B**) SQ22536. Effect of (**C**) forskolin or (**D**) 8-Br-cyclic AMP on I_K_. (**E**) Bar graph summarizing current density changes for the effects of SNAP on I_K_ after pre-incubation with KT5720 or SQ22536, and the effect of forskolin or 8-Br-cAMP on I_K_. * *p* < 0.05, ** *p* < 0.01 versus the control.

We examined the contribution of cAMP to SNAP-induced I_K_ enhancement, using KT5720 (a PKA blocker). When the cells were pretreated with KT5720 (1 µM) in the bath solution for 20 min, 100 µM SNAP did not increase the I_K_ (−1.1 ± 9.8% of the control, *n* = 7, Figure 6A). 

To further confirm the contribution of the cAMP signaling pathway, the cells were pretreated with SQ22536 (an AC blocker, 100 µM), 100 µM SNAP then failed to increase I_K_ significantly (+4.7 ± 11.3% of the control, *n* = 7, Figure 6B). On the other hand, forskolin (a stimulator of AC and an activator of PKA, 10 µM) increased the amplitude of I_K_ (+20.0 ± 11.0% of the control, *n* = 6, *p* < 0.05, Figure 6C). 8-Bromo-cyclic AMP (8-Br-cAMP, a cell-permeable cAMP, and an activator of PKA, 300 µM) also increased the activity of I_K_ (+40.4 ± 19.2% of the control, *n* = 5, *p* < 0.01, Figure 6D). The current densities at +30 mV with SNAP were not altered after KT5720 pretreatment (2.35 ± 0.49 pA/pF with control, 2.13 ± 0.42 pA/pF at KT5720, 2.33 ± 0.48 pA/pF with SNAP, Figure 6E) or after SQ22536 pretreatment (2.36 ± 0.88 pA/pF with control, 2.44 ± 0.97 pA/pF with SQ22536, 2.48 ± 0.99 pA/pF with SNAP). On the other hand, the current densities with the forskolin or 8-Br-cAMP effects on I_K_ increased significantly (2.49 ± 0.28 pA/pF with control, 2.99 ± 0.31 pA/pF with forskolin; from 2.44 ± 0.51 pA/pF to 3.42 ± 0.97 pA/pF with 8-Br-cAMP, Figure 6E).

### 2.6. Effect of NO on Delayed Rectifier K^+^ Current through the S-Nitrosylation Pathway

**Figure 7:**
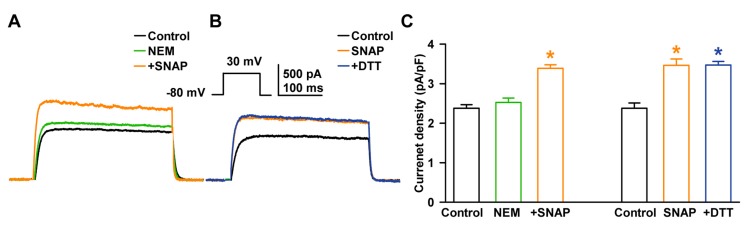
Influence of *S*-nitrosylation on the stimulating effect of NO on I_K_. (**A**) Representative currents showing the effect of SNAP on I_K_ after pretreatment with N-ethylmaleimide (NEM). (**B**) Effect of DL-dithiothreitol (DTT) on SNAP-stimulating I_K_. (**C**) Bar graph summarizing current density changes for SNAP effects on I_K_ after pretreatment with NEM and DTT. * *p* < 0.05 versus the control.

NO affects ubiquitous signaling pathways via posttranslational modification of cysteine residues, a reaction termed S-nitrosylation [28]. To examine the involvement of S-nitrosylation in SNAP-induced I_K_ enhancement, we pretreated the cells with N-ethylmaleimide (NEM, a thiol-alkylating reagent, 0.5 mM) and then applied SNAP (100 µM). In the presence of NEM, SNAP increased I_K_ significantly (+42.3 ± 10.1% of the control, *n* = 5, *p* < 0.05, Figure 7A), which suggests that the thiol residue was not the ultimate target of NO.

When DL-dithiothreitol (DTT, a reducing agent, 5 mM) was applied after I_K_ had been enhanced by SNAP (100 µM), it could not reverse the SNAP-induced enhancement of I_K_ (+45.5 ± 12.2% of the control with SNAP, *n* = 6, *p* < 0.05; +45.7 ± 6.9% of the control with DTT, *n* = 6, *p* < 0.05 for the control, Figure 7B). These findings suggest that *S*-nitrosylation is not the main mechanism for the NO stimulation of I_K_ in HCFs. Figure 7C shows the current densities with the SNAP effects on I_K_ after NEM pretreatment (2.38 ± 0.1 pA/pF with control, 2.53 ± 0.11 pA/pF with NEM, 3.39 ± 0.09 pA/pF with SNAP) and the current densities after DTT treatment following SNAP (2.38 ± 0.13 pA/pF with control, 3.40 ± 0.16 pA/pF with SNAP, 3.47 ± 0.09 pA/pF with DTT).

## 3. Discussion

In this study, we characterized the voltage-gated K^+^ (K_V_) channels in human cardiac ventricular fibroblasts and the effects of NO on the channels. We demonstrated the functional expression of two types of K_V_ channels: delayed rectifier K^+^ channel and transient outward K^+^ channel. We also observed that NO stimulated delayed rectifier K^+^ current (I_K_), but not transient outward K^+^ current (I_to_), through the sGC/cGMP/PKG pathway and AC/cAMP/PKA pathway, but not through *S*-nitrosylation. 

### 3.1. Identification of Two Types of Kv Channels in Human Cardiac Fibroblasts by Molecular Methods

The electrophysiological properties of cardiac cells are determined by the composition of ion channels and by their absolute abundance and proportional ratio. In healthy human hearts, the significant expression levels of K_V_1.2, K_V_1.5, K_V_1.7, K_V_2.1, and K_V_3.1 (for the I_K_) and K_V_1.4, K_V_3.3, K_V_3.4, K_V_4.1, and K_V_4.3 (for the I_to_) are found [31], which might indicate a functional role of these ion channel subunits in the formation of action potential in the human atrium and ventricle. K_V_1.5 is thought to be the major contributor to the I_K_ in human heart [23,32] and K_V_4.3 is responsible for the I_to_ [25,33,34]. However, very little is known about the expression of K^+^ channel subunits in human cardiac fibroblasts. 

In our RT-PCR analysis of mRNA expression of K_V_ channel genes in HCFs, there was a strong mRNA expression of K_V_1.1, K_V_1.2, K_V_1.5, and K_V_3.1 and a weak expression of K_V_1.6, K_V_1.7, and K_V_2.1 (for the I_K_). We also found that a strong mRNA expression of K_V_3.3 and K_V_3.4, and a weak expression of K_V_1.4, K_V_4.1, K_V_4.2, and K_V_4.4 (for the I_to_). 

These results differed from those of a previous report on K_V_ channels in HCFs [3]. In that report, the investigators found RT-PCR products corresponding to significant gene expression of K_V_1.5 and K_V_1.6 (for the I_K_) and K_V_4.2 and K_V_4.3 (for the I_to_), but found none for K_V_2.1 and K_V_3.1 (for the I_K_) or for K_V_1.4 (for the I_to_). The reason for the discrepancy in our results is unclear. 

The K_V_4.3 represents the predominant K^+^ channel subunit underlying I_to_ in human cardiomyocytes, as the most abundant K^+^ channel mRNAs were K_V_4.3 (80.7%) [35], but in our HCFs experiments, K_V_4.3 mRNA was not detected. To confirm our RT-PCR results for the K_V_ channels in HCFs, we repeated the RT-PCR several times to measure the gene expression of K_V_4.3 for the I_to_, but we found no gene expression. 

Changes in the expression of K^+^ channels explain the regional variations in morphology and duration of cardiac action potential among different cardiac regions and are influenced by heart rate, intracellular signaling pathways, drug and cardiovascular disorders [36]. Further investigations using molecular and electrophysiological approaches are needed to reveal the basis of ionic currents in cardiac fibroblasts in different heart regions because the extensive networks exist between cardiac fibroblasts and cardiomyocytes, that make the heterocellular electrical coupling [2,4,37,38,39]. 

### 3.2. Identification of Voltage-Gated K^+^ Channels in Human Cardiac Fibroblasts via Electrophysiological Methods

In our results, among the two types of K_V_ currents that could be distinguished by their activation and inactivation kinetics, I_K_ was the main subtype of K_V_ currents in HCFs, as I_K_ was recorded in 82.5% of HCFs with the whole-cell patch-clamp technique. This result was consistent with previous reports [11,40,41], but was somewhat discrepant with the results found by Li et al. [3].

According to their report on K^+^ currents in HCFs, IBK_Ca_ was present in most HCFs (88%), and I_K_ and I_to_ were equally present but in smaller populations (15% and 14%, respectively). 

The two types of K_V_ currents were 4-AP sensitive in HCFs and these results are consistent with others [15,42,43,44]. I_to_ was more sensitive than I_K_ to 4-AP, and similar results were found with human atrial I_to_ [35]. However, for TEA, another K^+^ channel blocker, the K_V_ currents showed different responses: a high concentration of TEA (10 mM) inhibited I_K_ (−42%) but not I_to_. In a study of human ventricular myocytes, I_K_ was not very sensitive to TEA: that is, the reduction was <20% with 10 mM external TEA [44]. On the other hand, 1 mM TEA could not inhibit either K_V_ current, consistent with previous reports: that is, low concentrations of TEA (≤1 mM) predominantly blocked Ca^2+^-activated K^+^ (K_Ca_) currents [45,46,47]. 

### 3.3. Effect of NO on Two Types of Voltage-Gated K^+^ Currents

We demonstrated that NO produced a concentration-dependent stimulation of I_K_ in HCFs, and these results were consistent with other reports in guinea-pig cardiomyocytes [24,48]. While a low concentration of NO (0.1–1 µM SNAP) did not affect the I_K_ of HCFs, a high concentration of NO (100 µM SNAP) increased the amplitude of I_K_ with an EC_50_ of approximately 26.4 µM in our experiment. In previous study, NO inhibited the hKv1.5 channel current, which generates I_K_ in transfected Chinese hamster ovary (CHO) cells and mouse ventricular myocytes [23]. 

This discrepancy could be explained by the concentration difference, because the effect of NO on cell function is determined by its concentration. Lower levels of NO (0.1–1 µM SNAP), which are observed under physiologic conditions, led to concentration-dependent inhibition of hKv1.5 current in cardiac myocytes [27], with the IC_50_ being approximately 340 nM [23]. Higher levels of NO (100 µM SNAP), which are observed in pathologic states, increased I_K_ [49,50]. 

We also demonstrated that NO could not affect I_to_ in HCFs; however, in previous study, NO inhibited K_V_4.3 in transfected CHO cells and I_to_ in human atrial and mouse ventricular myocytes in a concentration and voltage-dependent manner with an IC_50_ of 375 nM [25]. These differences could be explained by differences in the cell type and expressed K^+^ channel mRNA [36]. 

### 3.4. Effect of NO on Delayed Rectifier K^+^ Current through Protein Kinases Signaling Pathways and S-Nitrosylation

In our results, the stimulating effect of NO on I_K_ was suppressed in the presence of sGC inhibitor (ODQ) or PKG inhibitor (KT5823), and I_K_ was increased by 8-Br-cGMP, a cell-permeable analogue of cGMP. These results suggest that the stimulatory effects of NO are dependent on the sGC/cGMP/PKG pathway in HCFs. In previous reports, the cGMP-dependent pathway was suggested to play a principal role in NO action on hKv1.5-induced I_K_ in guinea-pig cardiomyocytes [24] and sinoatrial nodal cells [48]. 

We also investigated the effects of the AC/cAMP/PKA pathway on K_V_ currents in HCFs, because the PKA pathway is also responsible for NO-mediated cardioprotection [51], NO can directly activate AC and thereby increase cAMP levels [27], and AC activators or membrane-permeable cAMP analogs can increase NO production [52]. We found that specific PKA blockers (KT5720 or SQ22536) inhibited the stimulating effect of NO for I_K,_ and forskolin (an AC activator), and 8-Br-cAMP (a membrane-permeable cAMP) increased the currents in HCFs. 

These results indicate that NO (100 µM SNAP) increased I_K_ of HCFs through AC/cAMP/PKA signaling mechanisms. It has been reported that NO also modulates cardiac IBK_Ca_ channels, which are also important K^+^ channels in HCFs [13], and Na^+^ channels in guinea pig and mouse ventricular myocytes through the cAMP and cGMP pathways [53].

We also examined whether NO increased I_K_ through a cGMP-independent pathway, a direct *S*-nitrosylation of the thiol residue of target proteins, which is a well-known alternative pathway for the biological effects of NO [23,24,25,29]. *S*-nitrosylation regulates many cardiac ion channels and modulates the major currents involved in the generation of the action potential in cardiomyocytes [28]. Inwardly rectifying K^+^ current that determine resting membrane potential is also expressed in rat ventricular fibroblasts [3] and human ventricular fibroblasts [3]. NO increases the current by nitrosylation in cardiomyocytes and transfected CHO cells with Kir2.1 channel (the major isoform of inwardly rectifying K^+^ channel) [54]. 

It has been reported that *S*-nitrosylation increases I_K_ in guinea pig cardiomyocytes [55], but inhibits these currents in human atria [23]. However, in our results, the stimulating effect of NO on I_K_ was not blocked by NEM pretreatment in HCFs. In addition, the reducing reagent DTT did not reverse NO-induced I_K_ enhancement. These results suggest that *S*-nitrosylation is not involved in NO effects on I_K_ in HCFs. 

In summary, we provide evidence to demonstrate that NO modulates cardiac I_K_ but not I_to_ via a second messenger pathway through activation of PKG and PKA. This modulation occurs at a relatively high concentration of NO that was observed in pathological states. Therefore, it is reasonable to speculate that the modulation of I_K_ by NO may play a significant functional role in the pathological states. 

## 4. Materials and Methods

### 4.1. Cell Preparation and Culture

Commercially available primary adult human ventricular cardiac fibroblasts (HCF-av, Catalogue #6310 from ScienCell, Carisbad, CA, USA) were used. These cells were used for many bimolecular and electrophysiological experiments [3,13,40,56,57,58] and confirmed as fibroblasts by discoidin domain receptor 2 staining [58]. The cells were cultured in Dulbecco’s modified eagle’s medium (DMEM; Welgene, Gyeongsan, Gyeongsangbuk-do, Korea) that was supplemented with fetal bovine serum (10%, Welgene) and a penicillin-streptomycin solution (100×; GenDEPOT, Barker, TX, USA) in a humidified atmosphere of 5% CO_2_ and 95% air at 37 °C. Confluent fibroblasts were detached by incubation with trypsin (0.25%, Welgene) and ethylene diamine tetraacetic acid (0.02%) in DMEM for several minutes. The detached cells were pelleted by centrifugation, the supernatant was removed, and the pellet was suspended in 1 mL of bath solution. The cells used in this study were from early passages (3 to 7) to limit possible variation due to culture.

### 4.2. Reverse Transcription Polymerase Chain Reaction (RT-PCR)

mRNA was generated using the RT-PCR technique with the primers indicated in the Table 1 and Table 2. Total RNA was extracted from HCFs using the Total RNA Isolation PureLink RNA Mini Kit (Ambion, Carlsbad, CA, USA). First-strand cDNA was prepared with the SuperScript III Cells Direct cDNA Synthesis Kit (Invitrogen, Tokyo, Japan). Reverse transcription was performed in a S1000 Thermal Cycler (Bio-Rad, Hercules, CA, USA) according to the manufacturer’s instructions. RT-PCR reaction products (cDNA) were resolved by 1.2% agarose gel electrophoresis and stained with ethidium bromide for visualization under ultraviolet light. 

### 4.3. Electrophysiological Recordings

Recordings were obtained, using a whole-cell patch clamp with an Axopatch 200B Patch Clamp Amplifier (Axon Instruments, Union City, CA, USA) at room temperature. The pCLAMP 9.0 software (Axon Instruments) was used for data acquisition and analysis of whole-cell currents. Activated currents were filtered at 2 kHz and digitized at 10 kHz. Recording patch pipettes were prepared from filament-containing borosilicate tubes (TW150F-4; World Precision Instruments, Sarasota, FL, USA), using a two-stage microelectrode puller (PC-10; Narishige, Tokyo, Japan), and were then fire polished on a microforge (MF-830; Narishige). When filled with pipette solution, the pipettes exhibited a resistance of 2–3 MΩ. 

The bath solution to record K_V_ currents contained (in mM): 150 NaCl, 5.4 KCl, 1 CaCl_2_, 1 MgCl_2_, 10 glucose, and 5 HEPES (pH adjusted to 7.35 with NaOH). The pipette solution contained (in mM): 130 KCl, 1 CaCl_2_, 2 MgCl_2_, 10 HEPES, 10 EGTA, and 2 Mg-ATP (pH adjusted to 7.3 with KOH). TEA, 4-AP, SNAP, KT5823, ODQ, 8-Br-cGMP, KT5720, SQ22536, 8-Br-cAMP, and all other chemicals were purchased from Sigma-Aldrich (St. Louis, MO, USA). 

### 4.4. Statistical Analysis

Statistical analysis was performed using SPSS version 22.0 (SPSS Inc., Chicago, IL, USA). Results were presented as mean ± standard error of the mean (SEM). The paired Student’s *t*-test was used when appropriate to evaluate the statistical significance of differences between two group means, while one-way analysis of variance (ANOVA) was used for multiple groups. *p* values < 0.05 were considered statistically significant. 

## Figures and Tables

**Table 1:** Primers of delayed rectifier potassium channels used for RT-PCR.

Gene	Forward Primer	Reverse Primer	Size
GAPDH	5′-AGCCACATCGCTCAGACACC-3′	5′-GTACTCAGCGGCCAGCATCG-3	302
K_V_1.1	5′-CCATCATTCCTTATTTCATCAC-3	5′-CTCTTCCCCCTCAGTTTCTC-3′	488
K_V_1.2	5′-TCCGGGATGAGAATGAAGAC-3′	5′-TTGGACAGCTTGTCACTTGC-3′	747
K_V_1.3	5′-TCTGCCTATGCCCTTGTTTT-3′	5′-TTCCTCCCAGGATGTACTGC-3′	259
K_V_1.5	5′-TGCGTCATCTGGTTCACCTTCG-3′	5′-TGTTCAGCAAGCCTCCCATTCC-3′	906
K_V_1.6	5′-TCAACAGGATGGAAACCAGCCC-3′	5′-CTGCCATCTGCAACACGATTCC-3′	608
K_V_1.7	5′-CTTCCAGGGGCATGTTATTT-3′	5′-CTCAATGGAACTCAATTCAG-3′	300
K_V_2.1	5′-ACAGAGCAAACCAAAGGAAGAAC-3′	5′-CACCCTCCATGAAGTTGACTTTA-3′	383
K_V_2.2	5′-AACGAACTGAGGCGAGAG -3′	5′-ACTCCGCCTAAGGGTGAAAC-3′	546
K_V_3.1	5′-AACCCCATCGTGAACAAGACGG-3′	5′-TCATGGTGACCACGGCCCA-3′	550

**Table 2:** Primers of transient outward potassium channels used for RT-PCR.

Gene	Forward Primer	Reverse Primer	Size
GAPDH	5′-AGCCACATCGCTCAGACACC-3′	5′-ATCATTCAACAACCCACCAT-3′	302
K_V_1.4	5′-TGGCGGCTACAGTTCAGTCC-3′	5′-TGTTGACAATGACGGGCACAGG-3′	571
K_V_3.3	5′-TTCTGCCTGGAAACCCATGAGG-3′	5′-TGCCAAATCCCAAGGTCTGAGG-3′	694
K_V_3.4	5′-TTCAAGCTCACACGCCACTTCG-3′	5′-TTCTTTCGGTCCCGATAC-3′	656
K_V_4.1	5′-ATCTCGAGGAGATGAGGTTC-3′	5′-GATCCGCACGGCACTGTTTC-3′	318
K_V_4.2	5′-ATCTTCCGCCACATCCTGAA-3′	5′-GATCCGCACGGCACTGTTTC-3′	362
K_V_4.3	5′-GATGAGCAGATGTTTGAGCAG-3′	5′-AGCAGGTGGTAGTGAGGCC-3′	106
K_V_4.4	5′-AGCCAAGAAGAACAAGCTG-3′	5′-AGGAAGTTTAGGACATGCC-3′	315
